# The clustering of health behaviours in Ireland and their relationship with mental health, self-rated health and quality of life

**DOI:** 10.1186/1471-2458-11-692

**Published:** 2011-09-06

**Authors:** Mary C Conry, Karen Morgan, Philip Curry, Hannah McGee, Janas Harrington, Mark Ward, Emer Shelley

**Affiliations:** 1 Department of Psychology, Population Health Sciences, Royal College of Surgeons in Ireland, Dublin, Ireland; 2 School of Social Work & Social Policy, Trinity College Dublin, Ireland; 3 Department of Epidemiology & Public Health, University College Cork, Ireland

## Abstract

**Background:**

Health behaviours do not occur in isolation. Rather they cluster together. It is important to examine patterns of health behaviours to inform a more holistic approach to health in both health promotion and illness prevention strategies. Examination of patterns is also important because of the increased risk of mortality, morbidity and synergistic effects of health behaviours. This study examines the clustering of health behaviours in a nationally representative sample of Irish adults and explores the association of these clusters with mental health, self-rated health and quality of life.

**Methods:**

TwoStep Cluster analysis using SPSS was carried out on the SLÁN 2007 data (national Survey of Lifestyle, Attitudes and Nutrition, n = 10,364; response rate =62%; food frequency n = 9,223; cluster analysis n = 7,350). Patterns of smoking, drinking alcohol, physical activity and diet were considered. Associations with positive and negative mental health, quality of life and self-rated health were assessed.

**Results:**

Six health behaviour clusters were identified: Former Smokers, 21.3% (n = 1,564), Temperate, 14.6% (n = 1,075), Physically Inactive, 17.8% (n = 1,310), Healthy Lifestyle, 9.3% (n = 681), Multiple Risk Factor, 17% (n = 1248), and Mixed Lifestyle, 20% (n = 1,472). Cluster profiles varied with men aged 18-29 years, in the lower social classes most likely to adopt unhealthy behaviour patterns. In contrast, women from the higher social classes and aged 65 years and over were most likely to be in the Healthy Lifestyle cluster. Having healthier patterns of behaviour was associated with positive lower levels of psychological distress and higher levels of energy vitality.

**Conclusion:**

The current study identifies discernible patterns of lifestyle behaviours in the Irish population which are similar to those of our European counterparts. Healthier clusters (Former Smokers, Temperate and Healthy Lifestyle) reported higher levels of energy vitality, lower levels of psychological distress, better self-rated health and better quality of life. In contrast, those in the Multiple Risk Factor cluster had the lowest levels of energy and vitality and the highest levels of psychological distress. Identification of these discernible patterns because of their relationship with mortality, morbidity and longevity is important for identifying national and international health behaviour patterns.

## Background

It is well established that modification health related behaviours, can reduce mortality rates for all sections of the population [1]. While studies have documented the role of the 'big four' modifiable health behaviours (smoking, diet/nutrition, physical activity and alcohol consumption) separately in developing chronic illnesses, it is essential to consider patterns of health-related behaviours [2,3]. If a pattern of health behaviours is more prevalent than would be expected on the basis of marginal prevalence rates, the result is a cluster of health behaviours [4]. It has been found that while individual health behaviour patterns among Europeans have converged over time, reliable data on health-related risk factors is lacking to enable further international comparisons [5]. An exploration of clustering of health-related behaviours in a national population can contribute to planning of prevention and intervention strategies not only in national populations but also across Europe [4,6,7].

To date, studies identifying clusters in national populations across age groups have been limited [4,6,8], with studies focusing on either old [7] or young population samples [9]. A German study (n = 2,002) identified five homogenous clusters in the older population. One cluster was seen to represent an "ideal" health-related behaviour pattern; two clusters were smokers with problematic drinking patterns who had other unhealthy behaviours; and two clusters had a mix of healthy and unhealthy behaviours [10]. A Dutch study (n = 4,395) investigated the clustering of health-compromising and delinquent behaviours in adults and adolescents. It found that clusters differed between age group, with two clusters (Alcohol and Delinquency) for young adolescents and three clusters (Alcohol, Delinquency and Health) for older adolescents and adults [9].

Associations between clusters and mental health and other health outcomes have been found [11,12]. Health risk behaviours tend to co-occur in the population more frequently in those who are depressed. A French study (n = 17,355) found that those who were depressed were more likely to be daily smokers, have low fruit and vegetable intake and be cumulative risk takers [11]. On the other hand, co-occurrence of multiple healthy behaviours or protective health-related behaviours (being physically active, consuming five or more fruit and vegetable servings daily, being a non-smoker and moderate drinker) was associated with positive mental health, better self-rated health and healthier body weight [12].

Clusters of health behaviours are not randomly distributed in populations. Having multiple risk factors has been found to be more prevalent amongst women [8]. Clustering of health behaviours has also been found to be more pronounced at both ends of the spectrum, with more people than expected having all or none of the lifestyle risk factors. Chronic illnesses which are related to unhealthy clusters are documented as disproportionately represented in the lower social classes [8,13]. The clustering of unhealthy behaviours has also been found to have synergistic effects, which means that a combination of health behaviours is more detrimental to health than would be expected from the added individual effects of health behaviours, and this impacts on longevity [14,15]. Those with four risky health behaviours (smoking, excessive alcohol consumption, poor diet/nutrition and physical inactivity) have been found to die on average fourteen years younger than peers without these health behaviours [16].

Despite the identification of discernible patterns [4,6,8], related health outcomes and possible synergistic effects, many public health intervention strategies still focus on health behaviours in isolation. This approach while perhaps practical does not give adequate consideration to the fact that health behaviours do not occur in isolation but rather patterns of health behaviours exist. Similar to the proposed approach, the World Health Organization (WHO) has adopted a holistic approach to health which emphasises prevention by tackling combinations of modifiable risk factors.

As a result of the increased risk of synergistic effects, mortality and morbidity, examination of the clustering of health behaviours is important to support a more holistic approach to health in both health promotion and illness prevention strategies. International evidence indicates that health behaviours cluster and this indicates that a more integrated approach is required. This study establishes whether similar clusters of behaviours are identifiable in Ireland, and this information will inform the planning of prevention and intervention strategies not only in Ireland but also across Europe [4,6,7] This study aimed to identify how key health-related behaviours (physical activity, smoking, alcohol consumption and diet/nutrition) are distributed in a national population and to examine how these clusters compare to the findings of other studies. Furthermore, the study explored the relationship between the clusters and mental health, self-rated health and quality of life. This is the first study in the Irish context to identify clusters of behaviours and their relationship with mental health in a nationally representative sample.

## Methods

### General study design

As part of the third national Survey of Lifestyle, Attitudes and Nutrition 2007 (SLÁN) in Ireland, respondents were asked about their physical activity levels, alcohol consumption, smoking and diet/nutrition [17-19]. The GeoDirectory, which distinguishes between residential and commercial establishments of all addresses in the Republic of Ireland, was used as the sampling frame. The sample was a multi-stage probability sample, so each dwelling has a known probability of selection. It provided a cross-sectional, nationally representative sample of adults aged 18 years and over (n = 10,364 response rate: 62%) [18]. Full details on the sampling frame are available elsewhere [18]. A Willett Food Frequency Questionnaire was completed by 9,223 respondents [20]. As per the International Physical Activity Questionnaire (IPAQ) guidelines, extreme IPAQ values were removed from the dataset[21]. Therefore, for the purposes of the cluster analysis, there were 7,350 participants who had valid responses for the smoking, diet, physical activity and alcohol consumption questions. Weighting is not recommended for multivariate or cluster analysis, thus the unweighted percentages are reported.

#### Variables

##### Physical Activity

The International Physical Activity Questionnaire,(IPAQ) short form [21] was used to measure levels of physical activity, and responses were measured using the November 2005 scoring protocol [22]. IPAQ scoring provides continuous MET scores which can be classified into categories. In line with the November scoring protocol, participants were classified as follows: Low (little or no physical activity); Moderate (5 or more days of moderate intensity activity and/or walking of at least 30 minutes per day or specified equivalent, accumulating a minimum of 600 MET minutes/week); High (vigorous-intensity activity on at least 3 days and accumulating at least 1500 MET minutes/week or specified equivalent.

The IPAQ has been found to be a reliable measure which has been validated in a number of countries [22,23].

##### Alcohol Consumption

Drinking patterns were screened using the Alcohol Use Disorders Identification Test-Consumption (AUDIT-C)[24]. The AUDIT C has been widely used to assess drinking patterns [25,26] and in population studies [27,28]. Scores range from 0-12 where 0 indicates a non-drinker [29]. The cut-off point for moderate drinking adopted in this study was 5, which is in line with other European studies [30,31]. Respondents were classified using four categories: 0 (non-drinkers) and 1-5 (moderate drinking). Hazardous drinking patterns were classified as 6-8 (hazardous drinking) or 9-12 (very hazardous drinking)

##### Smoking

Respondents were asked if they smoked every day, some days or not at all and were then categorised as former, never or current smokers. Being a smoker was defined as 'having smoked at least 100 cigarettes during my lifetime'. 'Former smokers' were current non-smokers who had smoked at least 100 cigarettes in the past.

##### Diet

Diet was assessed with a Food Frequency Questionnaire (FFQ). This assesses the overall diet and included 150 food items arranged into the main food groups consumed in the Irish diet [20]. Respondents were then categorised according to their compliance with the Dietary Approaches to Stop Hypertension (DASH) advice: low salt intake, consumption of fruit and vegetables, and consumption of low fat dairy products. This diet has been shown to lower blood pressure and reduce cholesterol. The National Heart, Lung and Blood Institute promote DASH for treating hypertension, and it is promoted in the 2005 Dietary Guidelines for Americans [32]. Individuals' DASH score was ranked from 1-5 (1 = Poor, 2 = Fair, 3 = Good, 4 = Very good, 5 = Excellent).

##### Self-rated health

Self-rated health was measured using a single item. Respondents rated their overall health on a scale from 'excellent' to 'poor'. A single question on self-rated health is a valid and widely used measurement in European and International studies [33,34]. It is an established indicator of general health status and all-cause early mortality [35].

##### Quality of life

A question on quality of life, from the WHO's Quality of Life Survey [36] which has been used in several population studies [17-19], was used as an indicator of subjective well-being. This indicator is recommended when only a single question is used to assess quality of life (Power, 2003). Respondents were asked to rate their quality of life on a 5-point scale from 'very poor' (1) to 'very good' (5).

##### Mental Health

Positive and negative mental health was assessed using two subscales - Energy and Vitality (EVI) and Mental Health Index-5 (MHI-5) from the valid and reliable RAND SF36 [37-39]. The EVI measures both the occurrence and level of energy and vitality in the last month [39]. Respondents were asked to respond on a 6 category scale, going from 'All of the time' to 'None of the time' to 4 questions about affective aspects of their well being in the past month. Responses are presented as a sum score ranging from 0 to 100, with high scores indicating higher levels of energy and vitality [40,41]. The 5-item Mental Health Index-5 (MHI-5) measures levels of psychological distress during the last month. Responses are presented as a sum score ranging from 0 to 100, with low scores indicating higher levels of psychological distress [40,41]. To assist with the interpretation of regression coefficients both of these variables were rescaled by dividing by their inter-quartile range [42]. Rescaling has absolutely no effect on the magnitude of observed relationship, merely makes regression relationships easier to communicate.

##### Social Class

Social class was coded using the Irish Social Class schema. Individuals were categorised into groups based on similar levels of skill ranging from 1 (highest) to 7 (lowest). Individuals were then classified into six categories based on occupation category and employment status: SC 1-2 (professional and managerial); SC 3-4 (non-manual and skilled manual); SC 5-6 (semi-skilled and unskilled); and 'unclassified' [18].

### Statistical Analysis

Data was analysed using SPSS (Version 15.0). Clusters of health related behaviours were identified among 7,350 valid cases using the SPSS TwoStep Clustering algorithm. This algorithm is designed to efficiently handle large datasets, is capable of handling both continuous and categorical variables and has features to aid in determining the optimal number of clusters (SPSS, 2001). A further advantage of the TwoStep Cluster analysis approach is that it identifies which combinations are important from the many logically possible in the data and identifies the types empirically rather than impose them from an *a priori *scheme.

When analysing both continuous and categorical variables, TwoStep Clustering uses a model-based distance measure which defines the distance between two clusters as the corresponding decrease in log-likelihood by combing them together [43,44]. In the first step of the cluster analysis, the cases are sorted into pre-clusters. As SPSS examines a case it decides, based on the distance measure, whether a new cluster should be formed or if the case should be added to an existing cluster. The advantage of pre-clustering is that it reduces the size of the matrix which contains the distance between all possible pairs of cases. The result is that the size of the distance matrix is now dependent on the number of pre-clusters as opposed to the number of cases. In the second step, pre clusters are clustered using a hierarchical clustering algorithm. The Bayesian information criterion (BIC) is then used to select the "best" cluster solution, with smaller values of the BIC indicating better models.

Naming of clusters is a subjective process and the clusters were named in a way which best represented the most notable findings in the data. It is argued that while naming the clusters makes presentation to the audience easier [45], it is difficult to encapsulate the level of difference of clusters between clusters with labels. Clusters were assessed to determine the best possible name to represent the defining characteristics of individual clusters. The clusters are not intended to be represented along a continuum.

A multi-nominal logistic regression was performed using SPSS 17.0 NOMREG procedure to predict the odd's ratios of cluster membership with 'healthy lifestyle' as the reference category. Categorical demographic predictors were gender, social class and age. Continuous mental health predictors were Energy and Vitality (EVI) and Mental Health Index-5 (MHI-5) from RAND SF36 [37-39]. Crude odds ratios were calculated for all predictors. A full logistic regression model was then produced which included all predictors. Odds ratios in the full model were adjusted for all other variables in the model. No interaction effects were considered. Goodness-of-fit for the adjusted model was assessed using a Likelihood Ratio chi-square test.

## Results

### Sample Characteristics

Over half of participants were women (51%). Over half (56%) were aged between 18 and 44 years. Social classes 1-2 and 3-4 accounted for the majority of the sample (69%). Nearly one fifth were non-drinkers, almost half were moderate drinkers (46%) and the remainder reported hazardous drinking patterns. Approximately half of participants were never smokers. Overall activity levels were moderate, (48%), with over one fifth reporting high activity levels (24%). After IPAQ scores were treating using the data processing guideline, the mean IPAQ score for the participants was 1,5713.4 and the maximum score was 14,940. Almost half reported a poor or fair diet (48%) and the remainder reported good, very good or excellent diet (see Table 1).

**Table 1:** Weighted Sample Characteristics

	Frequency	Weighted %
**GENDER **(n = 10,278)		

*Men*	5063	49%

*Women*	5215	51%

**AGE **(n = 10,277)		

*18-29 yrs*	2588	25%

*30-44 yrs*	3199	31%

*45-64 yrs*	2977	29%

*65+*	1513	15%

**SOCIAL CLASS **(n = 10,278)		

*SC1-2*	3227	31%

*SC3-4*	3869	38%

*SC5-6*	1649	16%

*Unclassified*	1533	15%

**AUDIT-C (Alcohol Use Disorder Identification Test-Consumption) **(n = 10, 252)		

0 (non drinkers)	1909	19%

1-5 (moderate drinkers)	4663	46%

6-8 (problematic)	2257	22%

9-12 (very problematic)	1423	14%

**DASH (Dietary Approach To Stop Hypertension) **(n = 7,429)		

1 (poor)	1822	25%

*2 *(fair)	1700	23%

*3 *(good)	1191	16%

*4 *(very good)	1447	20%

*5 *(excellent)	1269	17%

**PHYSICAL ACTIVITY **(n = 10,051)		

Low	2873	29%

Moderate	4779	48%

High	2399	24%

**SMOKING **(n = 10,163)		

Former	1956	19%

Current	2888	28%

Never	5319	52%

Two Step Cluster analysis identified six distinct cluster groups with homogenous patterns of health-related behaviours. Of the 7,350 participants, 21% (n = 1564) were classified as Former Smokers, 15% (n = 1,075) as Temperate, 18% (n = 1310) as Physically Inactive, 9% (n = 681) as Healthy Lifestyle, 17% (n = 1,248) as Multiple Risk Factor and 20% (n = 1,472) as a Mixed Lifestyle.

### Cluster Profiles: Description, socio-demographic profile, mental health and well being

This section will detail the characteristics of each cluster and identify the specific group of people who were most likely to be in particular cluster. Table 2), the Healthy Lifestyle cluster was identified as the reference category. See Additional File 1.

**Table 2:** Crude and Adjusted odds ratios and 95% confidence intervals for risk associated with lifestyle cluster group membership.

Variable		Temperate				Former smokers				Mixed lifestyle				Physically inactive				Multiple risk factors			
		
		Crude		Adjusted		Crude		Adjusted		Crude		Adjusted		Crude		Adjusted		Crude		Adjusted	
		OR	95% CI	OR	95% CI	OR	95% CI	OR	95% CI	OR	95% CI	OR	95% CI	OR	95% CI	OR	95% CI	OR	95% CI	OR	95% CI
*Sex*	Female	1		1		1		1		1		1		1		1		1		1	
	Male	1.50**	1.20-1.87	1.68**	1.32-2.12	3.53**	2.88-4.34	3.63**	2.92-4.51	3.12**	2.59-3.92	3.85**	3.07-4.82	2.16**	1.75-2.67	2.39**	1.91-3.01	2.78	2.25-3.43	3.21**	2.55-4.04
																					
*Social class*	SC5-6	1		1		1		1		1		1		1		1		1		1	
	SC3-4	.82	.63-1.05	.69*	.49-.97	.99	.77-1.27	.66*	.48- .90	.64**	.51-.82	.60*	.43-.82	.72*	.57-.93	.47**	.34-.64	.80	.63-1.03	.47**	.34- .64
	SC1-2	.80	.62-1.04	.63*	.47-.93	1.05	.81-1.34	.71*	.51- .97	.45**	.35-.58	.41**	.30-.57	.58**	.45-.75	.38**	.27-.53	.49**	.38-.63	.29**	.21- .40
																					
*Age*	Over 65	1		1		1		1		1		1		1		1		1		1	
	45-64 yrs	1.14	.88-1.48	1.20	.90-1.61	.97	.76-1.23	1.00	.77-1.31	.65*	.51-.84	.70*	.53-.92	1.70**	1.30-2.24	1.77**	1.30-2.41	1.55*	1.17-2.05	1.56*	1.14-2.13
	30-44 yrs	1.48*	1.31-1.94	1.72**	1.128-2.33	1.05	.82-1.34	1.16	.88-1.53	.88	.69-1.14	1.10	.82-1.46	2.74**	2.07-3.62	3.33**	2.44-4.56	2.60**	1.95-3.40	3.22**	2.35-4.41
	18-29 yrs	1.64*	1.15-2.34	1.73*	1.15-2.61	.96	.69-1.35	1.28	.87-1.88	2.75**	2.00-3.77	3.33**	2.30-4.80	4.73**	3.35-6.68	5.92**	3.98-8.79	5.33**	3.78-7.53	7.38**	4.98-10.94
																					
*Psychological Distress*	MHI-5 (RAND SF36)	..83*	.74-.93	.87	..76-1.01	.89*	.80-.97	.97	.84-1.11	.78**	.70-.87	.78*	.68-.90	.72**	.64-.80	.76**	.66-.87	.64**	.58-.71	.77*	.67- .89
Energy and Vitality	EVI (RAND SF36)	.84*	.74-.96	.89	.75-1.06	.85*	.75-.96	.78*	.66-.91	.89	.78-1.01	.96	.81-1.13	.80**	.70-.90	.90	.76-1.06	.64**	.56-.72	.68**	.58- .80

Odds ratios are adjusted for all other variables in the model and the reference category is 'Healthy life style'.

OVERALL ADJUSTED MODEL STATISTICS: χ^2 ^(40) = 863.37***; Nagelkerke R^2 ^= .131; Cox and Snell R^2 ^= .128

* = p < .05 level ** = p < .001 level

**The Healthy Lifestyle cluster **(n = 681, 9.3%) reported relatively high levels of physical activity (IPAQ = 1544.98; high), were never smokers and had an excellent diet with all members scoring a DASH diet score of 5, representing the majority amongst the clusters. The majority were moderate drinkers (scoring 1-5) (67%), while one third were non-drinkers. Compared to the other clusters, individuals reported the highest levels of energy vitality (69.9), lowest levels of psychological distress (84.8), highest percentage with 'excellent' or 'very good' health (64.7%) and 'good' or very good' quality of life (91.6%). Compared to the other clusters, individuals in the Healthy Lifestyle cluster were more likely to be women, aged 65 years and over in the highest social class and report lower psychological distress.

**The Former Smokers cluster **(n = 1,564, 21%) accounted for 98% of former smokers in the population, reported the highest physical activity levels (mean IPAQ = 2569.74; high). Over half were moderate drinkers (scoring 1-5) and over 40% had a healthy diet. Individuals reported levels of energy and vitality (67.7) similar to the population average. Individuals reported above average low levels of psychological distress. (83.5). The percentage who reported 'excellent' or 'very good' health self-rated health and 'good or very good' quality of life was the same as general population proportions. Compared to the Healthy Lifestyle cluster, former smokers tended to include far more men (Adjusted OR = 3.63) and fewer members of the highest social class grouping (Adjusted OR = .71). There were no significant differences in age or psychological distress.

**The Temperate cluster **(n = 1,075, 14.6%) comprised moderately active (IPAQ = 1322.71; moderate), never smokers and moderate drinkers (scoring 1-5). DASH diet scores were mainly healthy but there were no high scores. Individuals reported levels of energy vitality (67.5) and psychological distress (82.4) similar to the population average. The percentage of those who reported 'excellent' or 'very good' self-rated health (60%) was second highest amongst the clusters, and the percentage reporting 'good' or 'very good' quality of life, 92% was highest compared to the other clusters. Compared to the Healthy Lifestyle cluster, the Temperate included more men (Adjusted OR = 1.63), fewer people of the highest social class (Adjusted OR = .63) and more in the age groups 18-29 years (Adjusted OR = 1.73) and 30-44 years (Adjusted OR = 1.72).

**The Physically Inactive cluster **(n = 1,310, 18%) reported the lowest levels of physical activity (IPAQ = 1131.19; moderate). Over half (54%) were current smokers and 41% reported hazardous drinking patterns (scoring 6-12). The majority (76%) had poor DASH diet scores. Individuals reported levels of energy and vitality (66.7) below the general population average and higher levels of psychological distress (80.2), which were the highest of all the clusters. The percentage reporting 'excellent or very good' self-rated health was 60% and 'good' or 'very good' quality of life was 88%. Compared to the Healthy Lifestyle cluster, individuals in this cluster were more likely to be men (Adjusted OR = 2.39), far more likely to be aged 18 to 29 years (Adjusted OR = 5.92) and far less likely to be in the highest social class (Adjusted OR = .38). Individuals in this cluster were also far more likely to report higher psychological distress.

**The Multiple Risk Factor cluster **(n = 1,248, 17%) reported moderate physical activity levels (IPAQ = 1233.20; moderate). The majority were current smokers (98%). Drinking patterns were mixed with nearly 40% moderate drinkers (scoring 1-5) and over 40% problem drinkers (scoring 6-12). DASH diet scores were varied, with over half reporting the lowest diet score and no representation from this cluster in the highest score category. Compared to the other clusters, individuals reported the lowest levels of energy and vitality (63) and highest levels of psychological distress (78.2). This cluster had the lowest percentage reporting 'excellent' or 'very good' self rated health (49%) and 'good' or 'very good' quality of life (84%). Scores on all the mental health and social well-being measures were below the general population average.

Compared to the Healthy Lifestyle cluster, individuals in this cluster were far more likely to be men (Adjusted OR = 3.21) and in the age group 18 to 29 years (Adjusted OR = 7.38). They were far less likely to be in the highest social class (Adjusted OR = .29) and more likely to report higher psychological distress and lower energy and vitality.

**The Mixed Lifestyle cluster **(n = 1472, 20%) were all never smokers who reported some physical activity (IPAQ = 1134.51; moderate). Over half reported poor diet. While over half (54%) were non-drinkers (scoring 0), almost half (46%) were problem drinkers (scoring 6-12). Levels of energy and vitality (68) and psychological distress (81.6) were similar to population levels. Similarly, the percentage of individuals who reported 'excellent' or 'very good' self-rated health (59%) or 'good' or 'very good' quality of life (89%) were similar to the general population proportions. Compared to the Healthy Lifestyle cluster, individuals in this cluster were far more likely to be men (Adjusted OR = 3.21) and in the age group 18 to 29 years (Adjusted OR = 7.38). They were far less likely to be in the highest social class (Adjusted OR = .29) and more likely to report higher psychological distress and lower energy and vitality.

## Discussion

The current study identifies discernible patterns of health related behaviours in the Irish population. Using SLÁN 2007 data, six clusters of health-related behaviours were identified: Former Smokers, Temperate, Physically Inactive, Healthy Lifestyle, Multiple Risk Factor, and Mixed Lifestyle. Former Smokers (21%) accounted for the largest percentage of the Irish population while the Healthy Lifestyle accounted for the smallest (9%). Similar to findings in the Dutch population, nearly 20% of the population had three unfavourable health-related behaviours [4]. Healthier clusters (Former Smokers, Temperate and Healthy Lifestyle) reported higher levels of energy vitality, lower levels of psychological distress, better self-rated health and better quality of life. In contrast, those in the Multiple Risk Factor cluster had the lowest levels of energy and vitality and the highest psychological distress. Identification of these discernible patterns is important because of their relationship with mortality, morbidity and longevity [1,46].

The identification of clusters of health-related patterns in the Irish population is similar to the findings of other countries [7,8]. Health-related behaviours tend to cluster in specific patterns, which Poortinga (2006) argues might explain some of the various combinations of risk that have been found in other studies [6]. There were a similar number of clusters (n = 6) identified in the Irish population and in other European populations [11]. There is evidence to suggest that the number of clusters may differ based on age group, with van Nieuwehuijzen (2009) finding two clusters for young adults (12-15 years) in the Dutch population and three clusters for older adolescents (16-18 years) and adults (19-40 years).

Consistent with other countries, clustering at both ends of the spectrum was found, with people having all or none of the unhealthy health related behaviours. Individuals were found to have multiple unhealthy behaviours, with those in the Multiple Risk Factor and Physically Inactive clusters having multiple unhealthy behaviours [4]. The coexistence of healthy and unhealthy behaviours in other countries [8] was also confirmed in this study. A positive relationship was found between physical activity levels and hazardous alcohol consumption and a negative relationship was found between physical activity and propensity to smoke [7,8,14].

Contextualising our findings is challenging for a number of reasons, in particular, a lack of available data from other countries [5]. Cross-country comparisons are also difficult because of the use of different health behaviour measures, cut-off points and categorisations [6,8]. Furthermore, studies which have previously reported clustering have investigated biological risk risks [47]. Identification of clusters of health-related behaviour patterns in national populations have been relatively limited, with the majority of studies to date focusing on specific population subgroups, including those aged 12-40 years [9] and older people [10].

To date, research on the association between health-related behaviours and mental self-rated health and quality of life has been limited [11]. This study looked at the clusters in relation to mental health and well-being. As expected, individuals with healthier behaviour patterns [11] were more likely to report positive mental health and more positive perceptions of their health [12]. This study also found that a higher proportion of individuals who had healthy patterns reported better quality of life than those in an unhealthy cluster. Therefore, it is argued that future intervention strategies to promote healthier health-related behaviour patterns should note the interconnected nature of mental health and behaviour patterns. More research is needed to see if patterns of behaviours and the associated health outcomes change over time.

The results show that there are specific groups of the population who are more likely to adopt an unhealthy health-related behaviour pattern. In contrast to other studies, this study examined different age cohorts in the population. Those in the Healthy Lifestyle group were most likely to be women aged 65 years and over and least likely to be aged 18-29 years while those in the Multiple Risk Factor and Physically Inactive were most likely to be men aged 18-29 years. One fifth of those in the Physically Inactive cluster reported that they were inactive due to an injury/disability/medical condition, while 40% cited a lack of time as the main reason. The most commonly cited reason amongst all of the clusters for being physically inactive was a lack of time. This might explain why those aged 65 years and over were most likely to be in the Former Smokers cluster, with high physical activity levels. In contrast to other studies [8], clustering of unhealthy behaviours was more pronounced for men than women.

As expected, the lower social classes accounted for a disproportionate share of those in the Physically Inactive cluster. Social classes 1-2 were the least likely of the social classes to fall into this cluster. Social classes 5-6 were the most likely of the social classes to be in the Physically Inactive or Multiple Risk Factor clusters. In contrast, social classes 1-2 were the most likely to be in the Temperate or Health Lifestyle clusters. Consistent with other studies, women were more likely than men to have no risk factors.

The findings of this study must be viewed in light of methodological considerations. First, only 7,350 responses of a potential 9,223 possible responses were eligible for inclusion in this study. Second, the data used in this study is self-reported, so social desirability in responses may be an issue. Third, the design of SLÁN is cross-sectional, which means that the data only provides a snapshot of the patterns of health behaviours amongst the population. It also means that it not possible to establish whether a causal relationship exists between lifestyle patterns and mental health, self-rated health or quality of life.

## Conclusions

We conducted an examination of clusters, mental health outcomes, self-rated health or quality and life in a nationally representative population. We found that particular health-related behaviour patterns are cumulative in specific population subgroups, and this raises questions about health strategies. While a lack of data and different measurement of health behaviours makes comparisons difficult, the identified clusters were similar to those identified for European counterparts. It is suggested that countries adopt similar methods of assessing health behaviours to permit further examination of the existence of particular health behaviour clusters. This is underway through the European Health Information Survey. Furthermore, research is needed to establish whether a multifaceted intervention approach targeting specific health behaviour clusters is more effective than the current single risk factor approaches. Preventative policies should take a holistic view of health which recognises the co-occurrence of health-related behaviours, well-being and mental health.

## Competing interests

The authors declare that they have no competing interests.

## Authors' contributions

All authors read and approved the final manuscript. MC was the lead author and undertook the statistical analysis. KM supervised the paper and gave advice on paper structure. PC advised on the research approach and statistical analysis. HM was SLÁN Project Director and was the lead of the design team and delivery team for survey. She oversaw data analysis, write-up and interpretation of SLÁN and provided editorial contribution to this paper. JH provided specialist advice on diet analysis. MW undertook composite variable construction. ES provided methodological advice and interpretation of findings.

## Authors' Information

Mary Catherine Conry, Research Officer. Karen Morgan, Lecturer. Philip Curry, Lecturer. Mark Ward, Research Officer. Hannah McGee, Professor of Psychology. Janas Harrington, Researcher. Emer Shelley, Senior Lecturer

## Pre-publication history

The pre-publication history for this paper can be accessed here:

http://www.biomedcentral.com/1471-2458/11/692/prepub

## Supplementary Material

Additional file 1 **Full regression results**. This file contains detailed output of the multinomial regression analysis on the clusters. Click here for file
